# The relationship between patient safety culture and adverse events in Iranian hospitals: a survey among 360 nurses

**DOI:** 10.1186/s13037-023-00369-6

**Published:** 2023-07-26

**Authors:** Saeideh Moosavi, Mohammad Amerzadeh, Mohammad Azmal, Rohollah Kalhor

**Affiliations:** 1 Student Research Committee, School of Public Health, Qazvin University of Medical Sciences, Qazvin, Iran; 2 Social Determinants of Health Research Center, Research Institute for Prevention of Non-Communicable Diseases, Qazvin University of Medical Sciences, Qazvin, Iran; 3 School of Medicine, Bushehr University of Medical Services, Bushehr, Iran

**Keywords:** Patient safety culture, Adverse events, Hospital

## Abstract

**Background:**

Adverse events have become a global problem and are an important indicator of patient safety. Patient safety culture is essential in efforts to reduce adverse events in the hospital. This study aimed to investigate the status of the patient safety culture, the frequency of adverse events, and the relationship between them in Qazvin's hospitals in Iran.

**Methods:**

The present study is a descriptive-analytical study conducted in six hospitals in Qazvin, Iran, in 2020. The study population was nurses working in Qazvin hospitals. We collected data via a patient safety culture questionnaire and an adverse event checklist. Three hundred sixty nurses completed questionnaires. Multiple logistic regression was used to investigate the relationship between variables.

**Results:**

The highest mean of patient safety culture was related to the organizational learning dimension (3.5, SD = .074) and feedback and communication about errors (3.4, SD = 0.82). The participants gave the lowest score to dimensions of exchanges and transfer of information (2.45,=0.86) and management support for patient safety (2.62,Sd = 0.65). Management's support for patient safety, general understanding of patient safety culture, teamwork within organizational units, communication and feedback on errors, staff issues, and information exchange and transfer were significant predictors of adverse events.

**Conclusion:**

This study confirms patient safety culture as a predictor of adverse events. Healthcare managers should provide the basis for improving the patient safety culture and reducing adverse events through methods such as encouraging the reporting of adverse events and also holding training courses for nurses.

## Background

Patient safety is the heart of healthcare quality [1]. It has received particular attention in the health sector of developed and developing countries in the last decade. The ongoing events that harm patients and the growing complexity of healthcare systems have intensified the need to provide safe care to patients, employees, and society [2]. Adverse events are common due to individual errors and system weakness in providing health service, which has become a global problem [3, 4]. The rate of adverse events is one of the critical indicators of patient safety. Adverse events are unintentional or unexpected incidents which hurt patients and might result in temporary or permanent disability [5]. A systematic review found the rate of adverse events between 7 and 40%. The most frequent events were complications associated infections, surgical procedures and medication. Another systematic review also showed that approximately 10% of patients were affected by adverse events and 43% were preventable, and 7.4% were fatal [6]. The rate of reporting adverse events in a study in Iran for a blood infection, pressure ulcer, patient fall, and hospital infection was 76.1%, 66.2%, 59%, and 57.7% respectively [7]. Studies have estimated 0.9 to 5.2% of hospital deaths as potentially preventable, equivalent to 1735, 11859, 210,000 to 400,000 per year in hospitals in the Netherlands, England, and the United States, respectively [8, 9].

Studies show that over 500,000 patients suffer from adverse events annulally in hospitals which might cost up to USD 5.6 million per hospital every year. The the additional expenditure of hospitalisation for patient with an adverse drug event is USD 2000 [10].

The American Institute of Medicine points out that preventable events occur due to poor skills and knowledge of nurses and doctors. It claims that system errors caused by problems in management, work environment, and employees are an important aspect of preventable adverse events [11]. Since individual errors are inevitable, the focus has changed from blaming individuals to improving systems [12]. Therefore, innovative solutions have been used to improve the systems, and one of these initiatives is to improve the patient safety culture [13].

The patient safety culture in healthcare organizations, especially hospitals, includes communications created based on mutual trust, good information flow, understanding of the importance of safety, organizational learning, management and leadership commitment, and the existence of a non-punitive approach to reporting errors and events [14]. Other countries, such as China, Palestine, Norway, and America have studied the relationship between patient safety culture and adverse events, which shows the importance and impact of patient safety culture on adverse events [15]. Studying this issue  in Iran with different burden of disese and context can add more evedince.

Although some studies in Iranian hospitals have evaluated the patient safety culture [16–19], the frequency of adverse events and the relationship between them and the patient safety culture have yet to be investigated. Besides, the starting point for creating a safety culture is to evaluate the current culture using a reliable and valid tool. Our hypothesis posits a significant association between the various dimensions of patient safety culture and the frequency of adverse events in healthcare. Our independent variable was patient safety and our dependent variable was adverse events. Therefore, this study aimed to investigate the relationship between patient safety culture and adverse events in Qazvin hospitals, Iran.

## Methods

The current study is a descriptive and analytical type conducted in six public hospitals in Qazvin city, Iran in 2020. The study population was nurses working in these hospitals. The inclusion criteria were: 1. at least one year of clinical service experience, 2. having a bachelor's degree, 3. full-time nurses, and 4. nurses who were willing to participate in the study. The exclusion criteria were: 1. Having less than one year of clinical service experience, 2. Less than a bachelor's degree, 3. Part-time nurses, 4. nurses who were not willing to participate in the study. Three hundred sixty nurses completed questionnaires.

We used a three-part questionnaire. The first part included demographic and organizational variables such as age, gender, marital status, education level, workplace department, and working hours per week. The second part was related to the evaluation of patient safety culture, which was evaluated using the standard hospital survey on patient safety culture questionnaire. This questionnaire was first designed in 2004 by the HealthCare Research and Quality Agency and has been used many times to evaluate patient safety culture in different parts of the world. Sora and Deir revised the questionnaire in 2010.

The questionnaire evaluates patient safety culture with 42 questions in 12 dimensions, including frequency of events reporting (3), general understanding of patient safety (4), management support for patient safety (4), organizational learning (3), teamwork within organizational units (4), the openness of communication channels (3), communication and feedback about errors (3), non-punitive response to error events (3), issues related to employees (4), management support for employees (3), teamwork between organizational units (4) and information exchange and transfer (4). The questionnaire also includes two questions about what score the respondents consider for patient safety in the ward and hospital and how many cases of reporting errors they had in the last 12 months. Iranian researchers translated this questionnaire into Farsi, confirming its validity and reliability in the study of Moghri et al. [20], whose Cronbach's alpha coefficient was between 57% and 80%.

In the current study, we studied the following six adverse events that often occur in the hospital, including pressure ulcers, patient falls, adverse drug events, surgical wound infection, infusion or transfusion reaction, and patients or their family complaints. We scaled the frequency of adverse events (0 = never, 1 = several times a year, 2 = once a month, 3 = several times a month, 4 = once a week, 5 = several times a week, 6 = every day).

The researchers went to the hospitals and provided the necessary explanations about the purpose of the study. Then the participants were assured that the study results would be published without their names and characteristics. The nurses were given a week to complete the questionnaires.

The sample size was determined by a confidence level of 95% and a variance of 0.5. The sample size of 384 nurses was obtained. Assuming response rate was 85%,therefore, 442 nurses were selected .A simple random sampling method was used to select nurses in the hospital

### Data analysis

We analyzed data by SPSS-22 software. Descriptive results related to patient safety culture, adverse events, and demographics are presented using mean, standard deviation, and percentage. Logistic regression was used to investigate the relationship between the level of patient safety culture and adverse events. For this purpose, six adverse events were divided into two groups (0 = never happened and 1 = happened) (such as several times a year, once a month, several times a month, once a week, several times per week, and every day). The confounding effect of other variables (age, gender, marital status, education level, work experience, workplace department, and working hours per week) was controlled in the model.

## Results

### Demographic findings

Among the 442 questionnaires, 390 questionnaires were returned (response rate = 88.2%), of which 30 questionnaires were incomplete and were excluded from the study. Finally, 360 questionnaires were included in the analysis (Table 1).

**Table 1:** Number of samples by hospitals

Hospital name	Number of nurses	Number of samples
Rajai	311	73
Kosar	189	41
Bu Ali	437	102
Ghods	212	50
Velayat	352	81
22 Bahman	58	13
Total	1560	360

Most nurses (48.8%) were in the age group of 31 to 40 years. Most were women (78.9%) and single (71.2%). Most participants had less than ten years of experience (78.9 percent) and a bachelor's degree (86.2 percent). Most of them were working in the general departments of the hospital (44.9 percent) and worked more than 44 hours a week (75.8 percent) (Table 2).

**Table 2:** Distribution of nurses based on demographic variables

Variable		Number	Percent
Age (years)	30 − 21	166	46.1
	40 − 31	134	37.2
	41≤	60	16.7
Gender	Male	54	15
	Female	306	85
Marital status	Single	118	32.8
	Married	242	67.2
Clinical experience (years)	10>	208	57.8
	10≤	152	42.2
Level of Education	Bachelor's degree	321	89.2
	Master's degree	39	10.8
Workplace department	Intensive care	109	30.3
	Emergency	23	6.4
	General sectors	228	63.3
The amount of working hours per week	≤ 44 hours	206	57.2
	> 44 hours	154	42.8
Total		650	100

The overall score of patient safety culture was, 3.06 with a standard deviation of 0.40, which indicates the average level of patient safety culture. Among the dimensions of patient safety culture, the highest mean was the organizational learning (3.0 ± 45.74), communication and providing feedback about errors (3.44 ± 0.82), and teamwork within organizational units (42.88 ± 0.3). Nurses gave the lowest score to the patient safety culture in information exchanges and transfer (2 ± 45.86), management support for patient safety (2 ± 62.65), and openness of communication channels (87 ± 83). 0.2) (Table 3).

**Table 3:** Mean scores of dimensions of patient safety culture from nurses' point of view

Dimensions of patient safety culture	Average	Standard deviation
Organizational Learning	3.45	0.74
Communication and feedback on errors	3.44	0.82
Teamwork within organizational units	3.42	0.88
Management support for employees	3.15	0.74
Events reporting frequency	3.14	0.82
A non-punitive response to an error event	3.10	0.93
Employees issues	3.05	0.76
Teamwork in organizational units	3	0.91
General understanding of patient safety	2.99	0.56
Open communication channels	2.87	0.73
Management support for patient safety	2.62	0.65
Exchanges and transmission of information	2.45	0.86
Overall patient safety	3.06	0.40

The score of patient safety culture in the department and hospital was 3.74 and 3.48, respectively, and the standard deviation was 0.83 and 0.87.

The most significant number of participants (48.1%) stated that they had never seen pressure ulcer scores in the past year. Most nurses (64.6 percent) never reported patient falls in the past year, and they never experienced adverse drug events (51.9 percent), surgical wound infection (51.5 percent), blood infusion or transfusion reaction (59.2 percent), and patients or their family complain (48.1%) (Table 4).

**Table 4:** The frequency of adverse events in the past year

	Never	Several Times A Year	Once A Month	Several Times A Month	Once A Week	Several Times A Week	Everyday
Pressure ulcer	(51.7%) 186	(32.18%) 118	(7.8%) 28	(4.7%) 17	(1.1%) 4	(1.1%) 4	(0.8%) 3
Patient fall	(66.9%) 241	(22.2%) 80	(7.2%) 26	(1.9%) 7	(0.6%) 2	(0.3%) 1	(0.8%) 3
Adverse Drug Events	(51.7%) 186	(37.5%) 135	(6.9%) 25	(2.8%) 10	(0.6%) 2	(0%) 0	(0.6%) 2
Surgical wound infection	(53.6%) 193	(31.1%) 112	(7.8%) 28	(5.6%) 20	(1.1%) 4	(.03%) 1	(0.6%) 2
Infusion or transfusion ‎reaction	(57.8%) 208	(30.3%) 109	(8.9%) 32	(1.7%) 6	(0.6%) 2	(0.3%) 1	(0.6%) 2
Patients or their family complaints	(48.9%) 176	(32.8%) 118	(5.3%) 19	(8.3%) 30	(0.8%) 3	(3.1%) 11	(0.8%) 3

Among the dimensions of patient safety culture, management support for patient safety had a significant effect on the incidence of bed sores (*P* < 0.001).

The results of the logistic regression model showed that the dimensions of communication and providing feedback about errors, issues related to employees and information exchange and transfer were significant predictors for patient falls (*P* < 0.05).

The dimension of teamwork among organizational units was the only dimension that had a significant relationship with the occurrence of side effects of the drug (*P* < 0.001).

The findings show that none of the dimensions of patient safety culture has a significant relationship with surgical wound infection.

Among the dimensions of patient safety culture, general understanding of patient safety culture was identified as a significant predictor of reaction to blood transfusion or injection (*P* = 0.03).

Among the aspects of patient safety culture, management support for patient safety was identified as a significant predictor of complaints from patients or their families (*P* = 0.01).

## Discussion

The present study investigated the relationship between patient safety culture and the adverse events based on nurses' point of view. Our findings show that management's support for patient safety, general understanding of patient safety culture, teamwork within organizational units, communication and feedback on errors, staff issues, and information exchange and transfer were significant predictors of adverse events.

The overall score of patient safety culture in the present study was at an average level. In line with our findings, Mustafaei et al. showed that the patient safety culture in Tehran's was 60 percent, at an average level compared to other countries. Among the different safety culture dimensions, the highest positive score was the teamwork dimension inside the hospital units and the frequency of adverse events [21].

According to our findings, among the dimensions of patient safety culture, the highest average was related to organizational learning, communication, and providing feedback about errors and teamwork within organizational units. In the studies of Nordin et al. and Singer et al., the lowest score of patient safety culture was the lack of management monitoring on patient safety [22, 23]. They believed that some patient safety challenges could not be solved with frontline healthcare providers; They should be considered at higher levels of organizations. In studies conducted in other countries such as Arabia, Turkey, Taiwan, Palestine, America, and Sweden, teamwork within the hospital units and organizational learning were the strengths of the patient safety culture, which is consistent with the our findings [24–27].

Al-Ahmadi et al. in Saudi Arabia [28] and Chen and Lee in Taiwan [29] found that among the dimensions of patient safety culture, the highest average was the dimension of organizational learning. They considered training and learning courses for their hospitals to increase understanding of patient safety culture and have emphasized the importance of teamwork. They believed they could create and strengthen the atmosphere of learning and cooperation in the organization and the spirit of the patient safety culture by putting the management of the patient safety culture on the agenda.

From the nurses' point of view, information exchange and transfer, management's support for patient safety, and openness of communication channels in the hospital were weaknesses. In a study conducted in Sweden by Danielson et al., management support for patient safety and employees were among the weaknesses of the patient safety culture [25]. In line with our findings, Elkuiz et al. revealed that the most critical weaknesses of the patient safety culture are the openness of communication channels, the exchange and transfer of information, and the non-punitive response to errors [24]. A review study in Arab countries conducted by Al-Montseri et al. also reported similar results [30]. Patient safety should be prioritized in hospitals and healthcare organizations because it harms patients and healthcare organizations. Communication is the main factor in the success of work and teamwork, especially among healthcare workers [30]. On the other hand, ineffective communication channels may lead to negative results, as reflected in low scores obtained in information exchange and transfer [31].

A study found that about 70% of nurses in Lebanon considered the dimensions of information exchange and transfer and management support for patient safety as the weakest dimensions of patient safety culture. They believed that the low level of these dimensions in the patient safety culture could deprive the nursing staff of strengthening the morale in critical conditions and handling a large amount of work [32]. The lowness of the above dimensions can lead to patient dissatisfaction with the hospital, which can be achieved by carefully managing the patient safety culture [33].

It is possible to strengthen the communication skills of nursing personnel and ward managers by conducting training courses for the dimensions of patient safety culture [34]. Based on the study conducted by Aiken et al. [35], the communication skills and support of managers in the studied countries were similar (USA 49.1%, Canada 43.4%, and Germany 32.6%), and managers' support for patient safety culture can significantly improve the dimensions of patient safety culture.

Our results showed that the prevalence of adverse events was high among nurses. Most nurses estimate that adverse events happen to them once a year. In the study of Ebadi et al. in Iran, the rate of reporting adverse events was between 57.7% and 76.1%. For blood infection, ‏patient fall, and hospital infection, 76.1%, 2 66.0%, 59%, and 57.7% have been reported [7]. In developed countries, the incidence of adverse events ranges from 3.5% in America, 9.2% in Canada to 12.3% in Sweden [36, 37]. Wang et al. in China showed that 47.8 to 75.6 percent of nurses estimated that various adverse events happened to them in the past year [38].

Based on our findings, patient safety culture is a significant predictor of adverse events; improving the patient safety culture will decrease adverse events in the hospital. Hassel et al. [35] showed that the relationship between patient safety culture and reporting of adverse events is direct and significant, so fewer adverse events were reported in hospitals with a higher patient safety culture. Another study showed an inverse relationship between patient safety culture and complications caused by the disease [39]. This result indicates that the higher the level of the patient safety culture, the fewer complications will occur.

## Rigor of study

Since this study was conducted retrospectively and the participants' information about adverse events during the past year was extracted, one limitation is recall bias, which can affect the accuracy of the information. Another limitation is the sensitivity of hospitals to adverse events and dealing with nurses and service providers, which can affect nurses' willingness to provide information.

## Conclusion

This study showed that nurses' understanding of patient safety culture was average, and the adverse events were high. Therefore, factors such as managers' support for patient safety in the hospital, nurses' understanding of the patient safety culture, investigating issues related to employees, and facilitating the exchange and transmission of information in the organization are necessary to reduce adverse events. This study reinforced the findings of previous studies that identified patient safety culture as a predictor of adverse events and introduced the promotion of patient safety culture as a factor in reducing adverse events. Further studies are necessary to determine the generalizability of these results in other environments and to identify interventions that reduce adverse events by promoting a patient safety culture. Healthcare providers should provide the basis for improving the culture of patient safety and reducing adverse events through methods such as encouraging the reporting of adverse events and holding training courses for nurses.

## Data Availability

The datasets used and/or analyzed during the current study available from the corresponding author on reasonable request. The entire dataset is in Farsi language. The Data can be available in English language for the readers and make available from the corresponding author on reasonable request.
